# GeNLP: a web tool for NLP-based exploration and prediction of microbial gene function

**DOI:** 10.1093/bioinformatics/btae034

**Published:** 2024-01-30

**Authors:** Danielle Miller, Ofir Arias, David Burstein

**Affiliations:** The Shmunis School of Biomedicine and Cancer Research, George S. Wise Faculty of Life Sciences, Tel-Aviv University, Tel-Aviv 6997801, Israel; The Shmunis School of Biomedicine and Cancer Research, George S. Wise Faculty of Life Sciences, Tel-Aviv University, Tel-Aviv 6997801, Israel; The Shmunis School of Biomedicine and Cancer Research, George S. Wise Faculty of Life Sciences, Tel-Aviv University, Tel-Aviv 6997801, Israel

## Abstract

**Summary:**

GeNLP is a web application that enables exploring microbial gene “semantics” and predictions of uncharacterized gene families based on their genomic context. It utilizes a pre-trained language model to uncover gene relationships and allows users to access and utilize the data as well as make their own predictions through an interactive interface.

**Availability and implementation:**

The web application is accessible from all browsers at: http://gnlp.bursteinlab.org/. All source codes are freely available from GitHub under the MIT license here: https://github.com/burstein-lab/genomic-nlp-server.

## 1 Introduction

Language models have emerged as a valuable tool for various biological data analysis tasks, including the analysis of genomic and protein sequences (Bepler and Berger 2021, Lin *et al.* 2023, Madani *et al.* 2023). These models are able to infer complex contextual patterns and relationships by processing large amounts of data allowing recent research to demonstrate their effectiveness in performing tasks, such as gene prediction, functional annotation, and gene family classification. By treating gene families as “words” and genomes as “sentences,” we have recently used Natural Language Processing (NLP) techniques to generate numerical representations of genes, termed “embeddings,” based on their genomic context, namely—the coding and non-coding regions that surround the gene itself. The genomic context of a gene holds important information about its function, particularly in prokaryotes, where co-localization of genes within the genome is correlated with co-function (Overbeek *et al.* 1999, Shmakov *et al.* 2018). As a result, genes that share a similar function are expected to have a shared genomic context. This means that these genes are likely to be located in close proximity to each other within the genome or have similar arrangements of regulatory elements and genetic features. Consequently, when we represent these genes using embeddings, which capture the semantic representation of their characteristics, we expect to observe similarities in their embeddings, reflecting their “semantic” resemblance. Thus, the embedding representations can be used to explore functional relationships between genes and predict multiple features, such as protein structure, gene function, and more (Bepler and Berger 2021, Jumper *et al.* 2021, Brandes *et al.* 2022, Miller *et al.* 2022, Lin *et al.* 2023).

Here, we present GeNLP, a web application aimed to allow users to explore the “semantic relationships” between thousands of microbial gene families without requiring any programming expertise. This web server is based on a model (Miller *et al.* 2022) adapted from word2vec (Mikolov *et al.* 2013), which was trained on all assembled metagenomes and genomes (excluding green plants, fungi, and animals) publicly available on NCBI’s GenBank Whole Genome Shotgun (WGS) database (Sayers *et al.* 2021) and all assembled metagenomes from EMBL-EBI’s MGnify database (Mitchell *et al.* 2020). From WGS genomes a representative subset based on Uniprot’s non-redundant (NR) proteomes was used to mitigate redundancies. Consequently, a substantial portion of the dataset primarily consists of metagenomic sequences for which taxonomic mappings are not known. As a preprocess, genetic data were clustered into 563 589 gene families based on sequence similarity and mapped to the KEGG orthology (Kanehisa 2019). However, due to the nature of the data, many gene families have no annotation in any existing database. Gene families with a significant hit to the KEGG dataset were assigned an identifier based on the KEGG Ortholog (KO) group they belong to, whereas gene families with no mapping were given a serial identifier. In order to maintain consistency, the KEGG proteins used for mapping KOs were subjected to subclustering, employing identical parameters utilized for clustering gene families without KO hits. This procedure aimed to refine and consolidate gene families based on sequence similarity, fostering a more precise representation. Genes that had sufficient occurrences in the genomic and metagenomic datasets to produce a meaningful context signature were included. The model was then extended to predict one of 10 major functional categories (namely, amino sugar and nucleotide sugar metabolism, benzoate degradation, energy metabolism, oxidative phosphorylation, porphyrin and chlorophyll metabolism, prokaryotic defense, ribosome, secretion systems, two-component systems, and other), based on KEGG’s functional ontology, taking into consideration KEGG’s hierarchy (Miller *et al.* 2022). The classification was performed using the pre-trained gene families embedding as input to a fully connected deep neural network. Prediction scores were derived from a combination of the softmax function-based classification score and the model’s accuracy in predicting the specific function, as measured by area under the precision–recall curve (AUPR). To ensure precision, a prediction was considered to have high confidence if it passed the threshold (*t* = 0.9) for the AUPR-weighted score and (*t*'=0.99) for the unweighted score.

Aiming to make the data and results of our NLP-based approach more accessible to the scientific community, we have developed GeNLP, a user-friendly web application that provides easy access and utilizes our model via an intuitive interface. Researchers can submit sequence data queries, run the model, browse through the embedding space, and view the results, all through their web browser. This convenient platform eliminates the need to execute the entire pipeline for gene prediction and simplifies individual analysis. Users can swiftly retrieve predictions assigned to their sequences by the model and further investigate genes that may be associated with them.

## 2 The GeNLP web application

**Figure 1.**
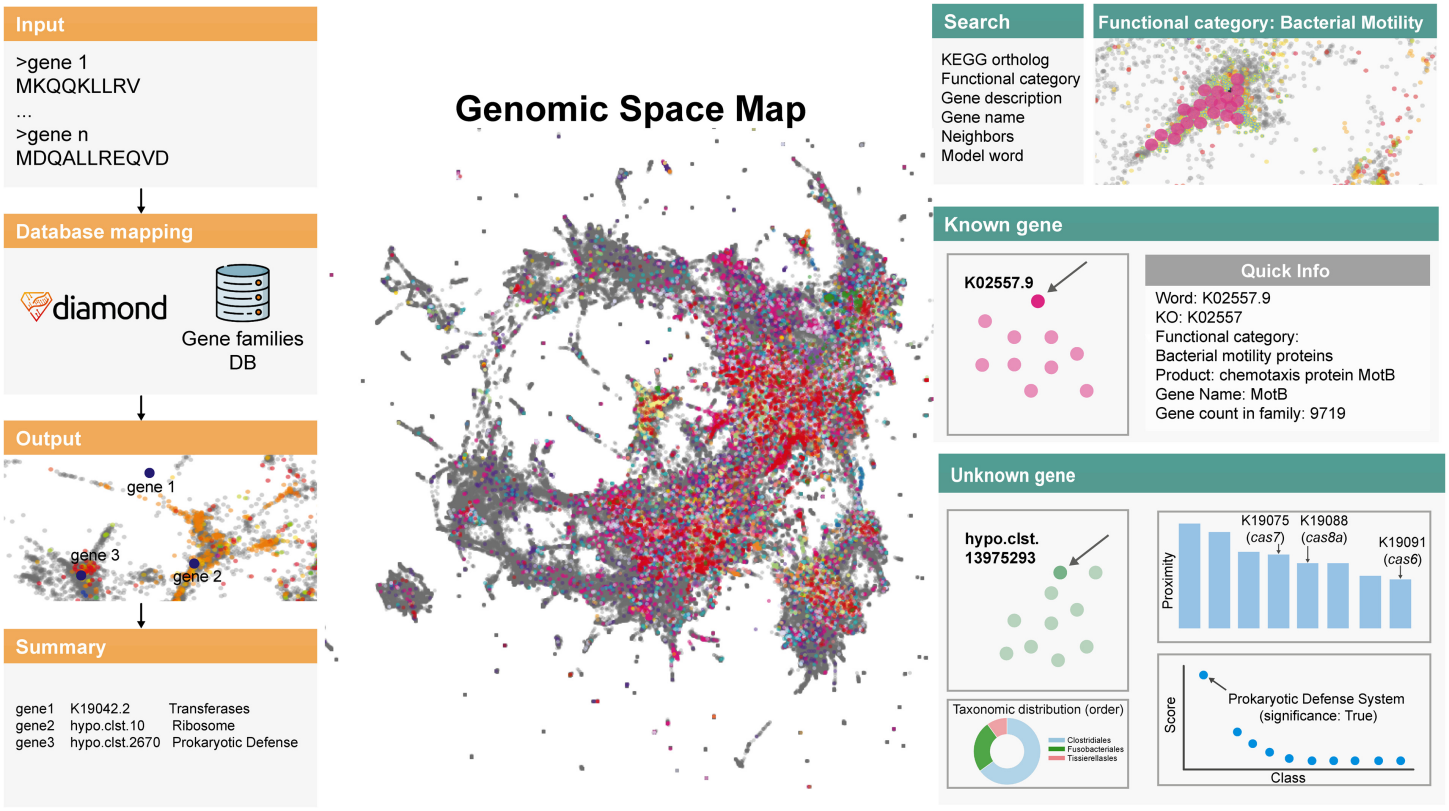
GeNLP overview. The left panel presents the sequence search mode workflow. The right panel demonstrates the explanatory mode features.

GeNLP is an interactive web application that enables the visualization and exploration of microbial gene families based on their genomic context. Utilizing our pre-trained language model (Miller *et al.* 2022), the platform allows users to uncover gene relationships and make predictions for unknown gene functions. The web service, available at http://gnlp.bursteinlab.org/, provides access to the data and functionality of the GeNLP application. The source code, implemented in Python, Vue, and TypeScript, is readily available on GitHub (https://github.com/burstein-lab/genomic-nlp-server). We offer a comprehensive, step-by-step tutorial, guiding users through the application, complemented by a concise demonstration, and provide this content both as Supplementary Material accompanying the manuscript and available on the web server’s wiki in its GitHub repository. To facilitate usage and maintenance, the web application has been implemented using Docker and requires no external installations. The GeNLP application offers two primary modes of operation: (i) a static explanatory mode for inspecting the gene embedding space and (ii) a custom predictive mode for querying user-provided data (Fig. 1). Through the application, users can delve into the complex network of genes, exploring both known and unknown genes, and gain a deeper understanding of their relationships and functions. For known genes, the application enables users to uncover genes with similar embedding and their functions, as well as locate multiple known genes that belong to the same KO group or share similar functions. Furthermore, the application can reveal genes with multiple “semantic meanings,” i.e. genes that have various genomic contexts and play a part in different pathways or complexes. For unknown genes, the GeNLP application offers the opportunity to explore their predicted functions by the pre-trained model (out of the abovementioned 10 functional categories) and provides their proximity in the embedding space to known genes that may share similar functions. In addition, each gene family contains information regarding its gene count and the taxonomic distribution of the genomes encoding for it. Users can also explore the spatial distribution of multiple selected genes in the gene embedding space. This allows for a more comprehensive analysis of the gene network and enables the discovery of new relationships and functions. Our application currently relies on gene contextual embedding computed using an adapted version of word2vec (Mikolov *et al.* 2013, Miller *et al.* 2022). As more informative approaches for gene representation are developed or major updates to the KEGG orthology database are released, the embedding space in the web application will be recomputed and the interface updated accordingly.

## 3 Gene exploration—gene space viewer

This mode offers a user-friendly and interactive approach to explore a vast genetic landscape comprising 563,589 gene families (representing a total of 360,039,110 genes), most of which are unannotated. The main layout presents a comprehensive map of gene families sourced from NCBI’s GenBank WGS and EMBL-EBI’s MGnify databases. The model’s context-based embedding, represented by a 300D vector, is projected into two dimensions for visualization using the UMAP algorithm. It is important to note that the 2D distances obtained through the UMAP dimension reduction (McInnes *et al.* 2020) may exhibit distortions compared to the original 300D cosine distances in the embedding space. Gene families are color-coded based on functional groups derived from the KEGG ontology or are in grey if they are not assigned to any KO group. Upon sufficient zoom-in, users can access an interactive mode, enabling them to obtain quick information about each gene, including its name, function, and description, by clicking on a point in the space. For genes lacking KEGG annotation, the predicted functional category obtained by our model is noted, along with its confidence, and annotation based on the NCBI NR database, if available. Additionally, the interactive mode offers several features per point: the gene count in the family, a “Neighbors” bar plot, a “Function prediction” dot plot, and a “Tax map” donut plot. The “Neighbors” bar plot displays the 10 closest genes in the high-dimensional embedding space, based on cosine distance, clicking on one of the bars will lead the user to the corresponding gene family. The “Function prediction” plot highlights for unknown genes the prediction scores across 10 functional categories as mentioned above. The “Tax map” depicts the taxonomic distribution at the order level within a gene family, based on genes from assembled genomes. The donut plot showcases the top 10 orders, including an “Other” category if the gene family is represented in more than 10 distinct orders. Given that a significant portion of genes originated from metagenomic databases that lack well-defined taxonomic origin, the plot indicates the percentage of genes within the gene family with a known taxonomic mapping in NCBI’s GenBank WGS database. The results displayed in the “Neighbors” and “Function prediction” plots, as well as a representative gene sequence, can be downloaded for each family.

For ease of use, this mode also enables the following six search modes, each allowing for direct typing or a dropdown menu with all options.

1. KEGG ortholog: searches by KO group identifier. Presents the gene family or families assigned to a KO.
2. Functional category: highlights specific functional groups as defined by the KEGG ontology. For example, selecting of “Prokaryotic Defense System” will present all known genes related to this functional category.
3. Gene description: searches genes according to their product description. This mode can be used only for annotated KOs with an existing description in KEGG.
4. Gene name: searches genes by their short name as assigned by KEGG.
5. Neighbors: given a gene family identifier, highlights the 10 closest gene families in the high-dimensional embedding space, obtained by the pre-trained model.
6. Model word: searches by KO subcluster or hypothetical identifier (used for unannotated genes). This information is derived directly from the corpus used to train the model, it includes all 563,589 gene families and can be utilized to follow-up investigations that were previously conducted on GeNLP.

### 3.1 Gene function prediction

The “Sequence” search mode allows users to submit a sequence query in FASTA format either by uploading a file or directly pasting protein sequences into the search box. If a family with sufficient similarity is found (*E*-value < 10^−4^), the web server will provide information for the best hits to the proteins provided, along with the *E*-values and percent identity. The sequences are mapped to our gene family database using the DIAMOND (Buchfink *et al.* 2015) tool and assigned an identifier based on the best hit satisfying the *E*-value threshold. If no such hit is identified, a message is displayed indicating that no significant hit was found for the given protein sequence. This might occur for rare microbial genes that are not well represented in genomic/metagenomic databases. When a significant hit is found, the identifier corresponding to the most similar gene in our database, which can be either a gene assigned a KEGG orthology or a cluster of unknown genes, is reported. It is important to note that this process is subject to the running time of DIAMOND, and may take several minutes, depending on the length and number of the submitted sequences. Once the predictions are generated, they, along with the corresponding gene family identifier and additional information, are available for downloading. These identifiers can later be used for self-exploration using the space viewer feature, as described in the previous section. This feature allows users to quickly and easily obtain predictions for specific genes and offers a convenient means of discovering genes with similar contextual signatures. The major advantage of this approach lies in its ability to provide insights into gene function that are not based on sequence homology but rather on the genomic context in which these genes are found across numerous organisms. Thus, users can infer function for genes lacking sequence similarity to any annotated gene. For genes with existing annotations, it facilitates rapid exploration of other genes with similar genomic context, and therefore, potentially similar functions. Furthermore, it enables the identification of previously unknown functions that a given gene may have according to the different genomic contexts in which it was found. Our approach broadens the scope of gene analysis, offering a more comprehensive understanding of gene functionalities beyond conventional sequence-based searches.

## Supplementary Material

btae034_Supplementary_Data

## Data Availability

No new data were generated or analyzed in support of this research.
