# Supplementary material for: GeNLP: a web tool for NLP-based exploration and prediction of microbial gene function
Source: Bioinformatics. 2024 Jan 30;40(2):btae034. doi: 10.1093/bioinformatics/btae034 (PMC10868303; doi:10.1093/bioinformatics/btae034)
Supplement: btae034_Supplementary_Data [file btae034_supplementary_data.pdf]

GeNLP: a web tool for NLP-based exploration and prediction of microbial gene function

Supplementary Material

Danielle Miller[1], Ofir Arias[1], and David Burstein[1]

[1]The Shmunis School of Biomedicine and Cancer Research, George S. Wise Faculty of Life Sciences, Tel Aviv University, Tel Aviv, Israel
davidbur@tauex.tau.ac.il, daniellem1@mail.tau.ac.il, ofirarias@gmail.com

Visit the GeNLP website: https://gnlp.bursteinlab.org

The server is based on a pre-trained published model: "Deciphering microbial gene function using natural language processing" https://www.nature.com/articles/s41467-022-33397-4

Weights and trained model are available on the paper's GitHub repository. https://github.com/burstein-lab/genomic-nlp

This is a quick user guide for the GeNLP website. It is also available under the GitHub wiki https://github.com/burstein-lab/genomic-nlp-server/wiki.
The Application contains two main modes:

- Explanatory Mode
- Predictive Mode

In the following pages, you will find a comprehensive, step-by-step demonstration of how the website functions.

Explanatory Mode

The map in the main display is an interactive map, where each gene is represented by a dot. The map is color-coded by functional group, where unknown proteins are colored in light grey. The map supports zoom-in and zoom-out. Upon sufficient zoom-in the points are clickable, providing additional information on a given gene family.

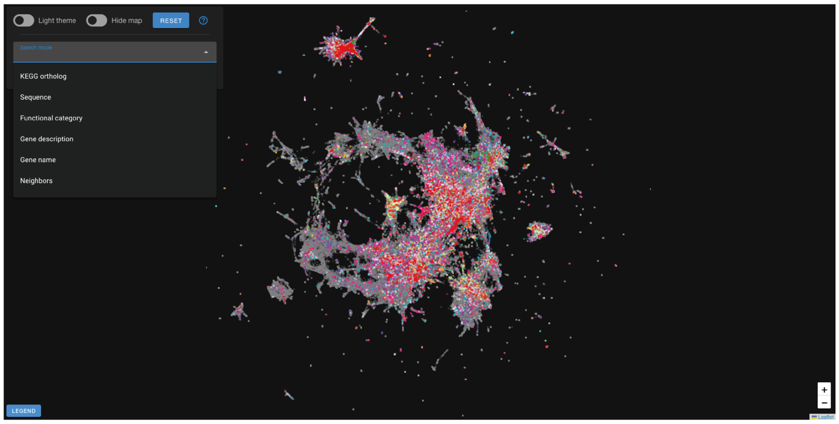

Supplementary Fig. 1: GeNLP home screen

The data points are color-coded based on their functional groups, which have been adapted from the KEGG (Kyoto Encyclopedia of Genes and Genomes) database. A legend depicting these functional groups is located in the bottom left corner of the visualization.

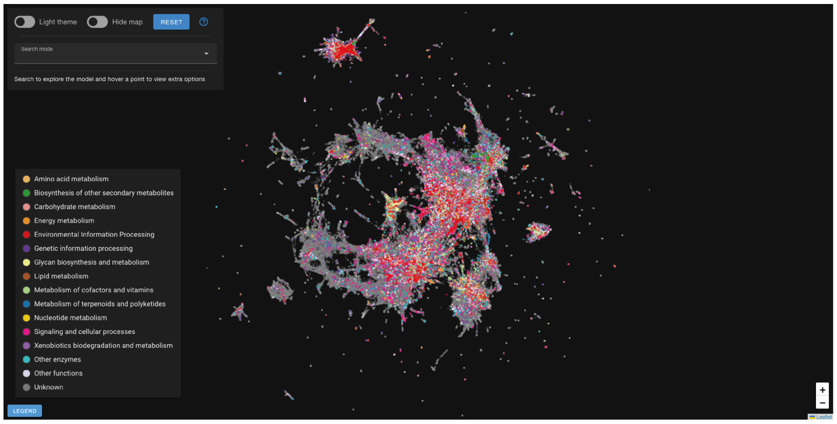

Supplementary Fig. 2: Gene family color-code legend, the category will be highlighted upon selection

KEGG ortholog

Search by KEGG ortholog group identifier (KO). For example, the KO **K07464** (*cas4*), The yellow circles mark the location of the highlighted interactive points of *cas4* representatives.

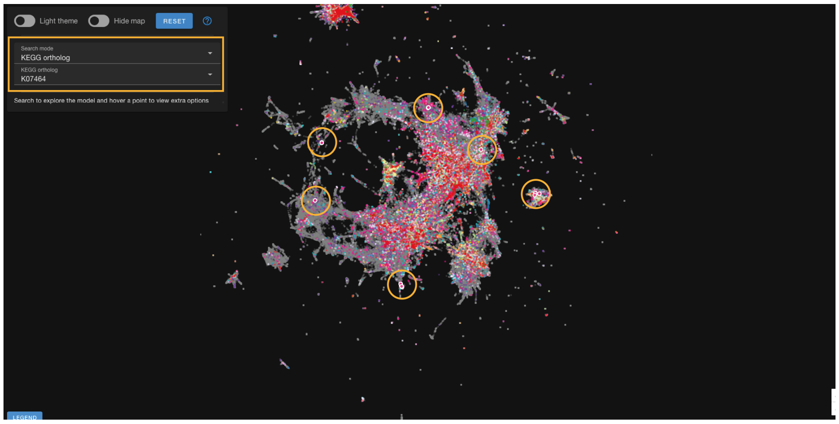

Supplementary Fig. 3: KEGG ortholog example (K07464)

Functional category

Highlight a specific functional category. For example, selecting **Bacterial Motility Proteins** will result in all related proteins being highlighted as interactive points

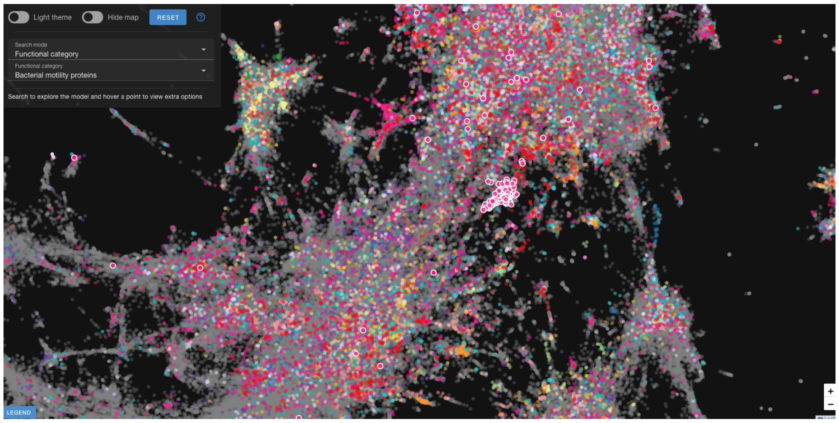

Supplementary Fig. 4: Functional category example ("Bacterial Motility Proteins")

Gene description

Highlight genes sharing the same gene description. For example, selecting **CRISPR system Cascade subunit casB** will result in all related proteins being highlighted as interactive points.

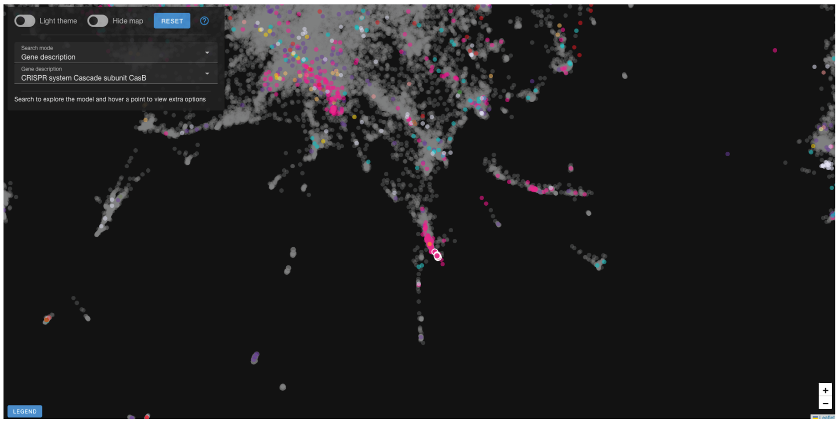

Supplementary Fig. 5: Gene description example (”CRISPR system Cascade subunit casB”)

Gene name

Highlight genes according to their gene name. For example, selecting *cas3* will result in all related proteins being highlighted as interactive points. In this detailed zoom-in resolution, all points are interactive, and the selected points are distinctly highlighted with a white edge color.

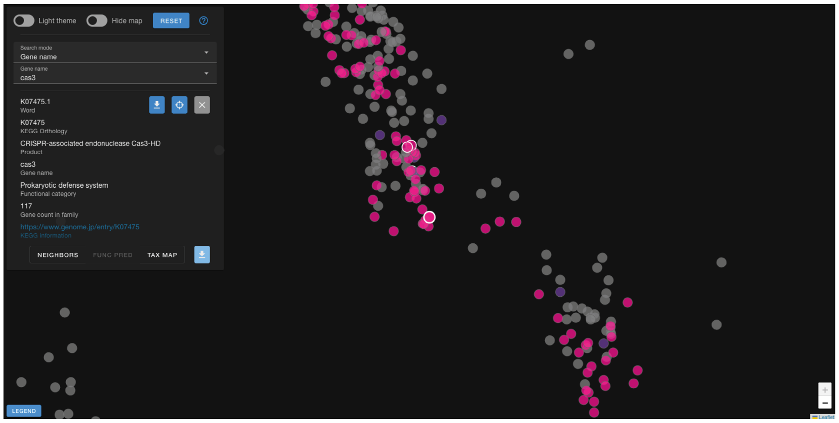

Supplementary Fig. 6: Gene name example (cas3)

Neighbors

Highlight the 10-closest genes for a selected gene family. For example, selecting the word **CRISPR** (which corresponds to a CRISPR array identifier will highlight its neighbors as interactive points.

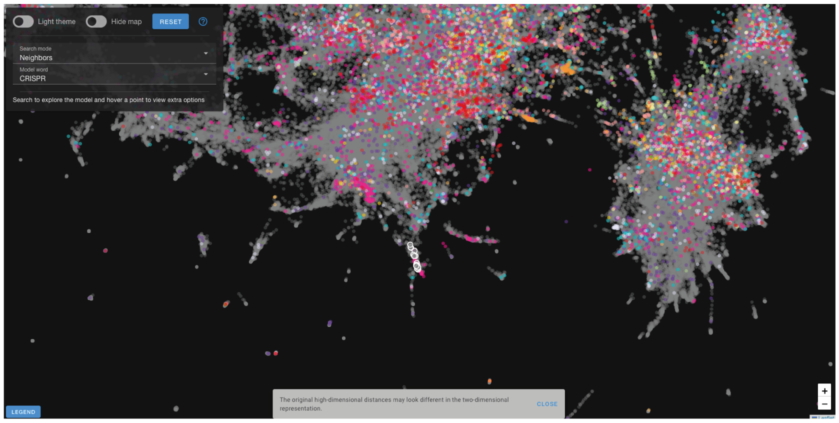

Supplementary Fig. 7: Neighbors example (CRISPR)

Notice: The distance calculations were performed in a 300-dimensional space. As a result, it is possible that the genes closest to each other may appear to be far apart in the two-dimensional projections. A banner noting this appears when entering the web application, and again when performing a search for neighbors.

Model word

Search by KEGG ortholog group (KO) sub-cluster or by a hypothetical identifier (used for uncharacterized genes). Multiple selections are supported. The aforementioned description also applies when selecting points in interactive mode.

When selecting a specific word (point), we offer relevant information about the associated gene, which may vary depending on whether the gene is known or unknown. Furthermore, we provide two interactive panels: **NEIGHBORS**, **FUNC PRED**, and **TAX MAP**, offering additional interactive functionalities for further exploration and analysis.

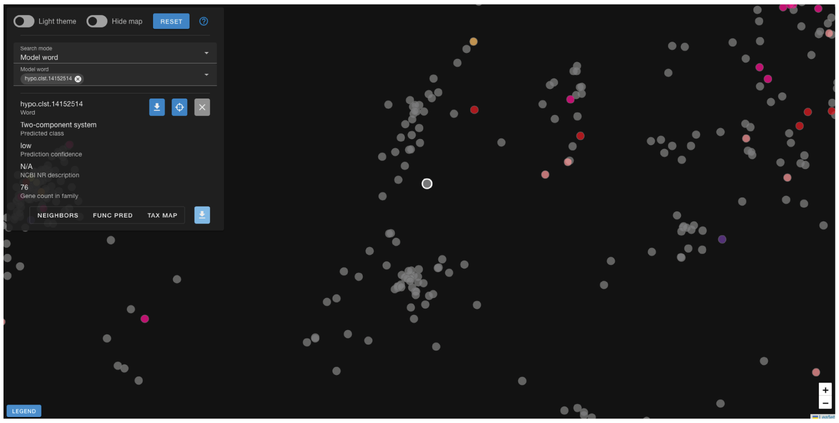

Supplementary Fig. 8: Model word example (hypo.clst.14152514)

For hypothetical proteins, the field *Prediction confidence* denotes whether the prediction assigned by the model is reliable. The score is obtained by the functional classifier, high prediction confidence stands for a score that passed our defined cutoff, whereas low prediction confidence is for scores below the cutoff (for more technical details see the manuscript).

Upon selecting the **NEIGHBORS** tab, a graph displaying the ten closest gene families will be presented. Clicking on a specific neighbor within the graph will trigger a zoom-in effect on the corresponding neighboring genes, providing a more detailed view.

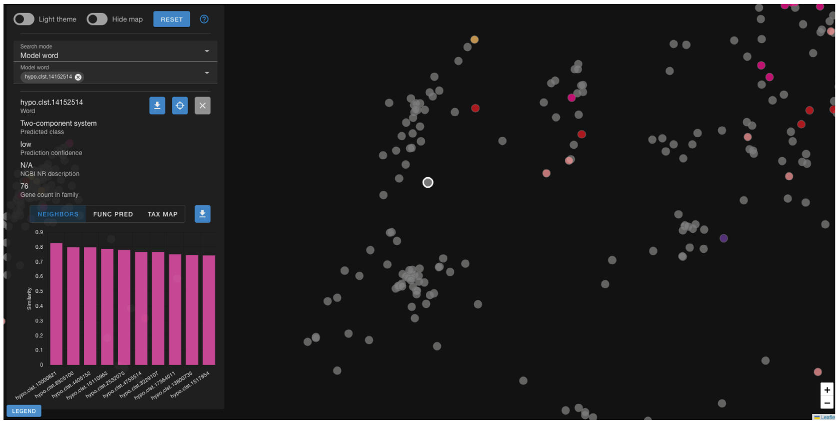

Supplementary Fig. 9: Model word NEIGHBORS field (hypo.clst.14152514)

Upon selecting the **FUNC PRED** tab, a graph displaying the prediction score per inspected category will be presented.

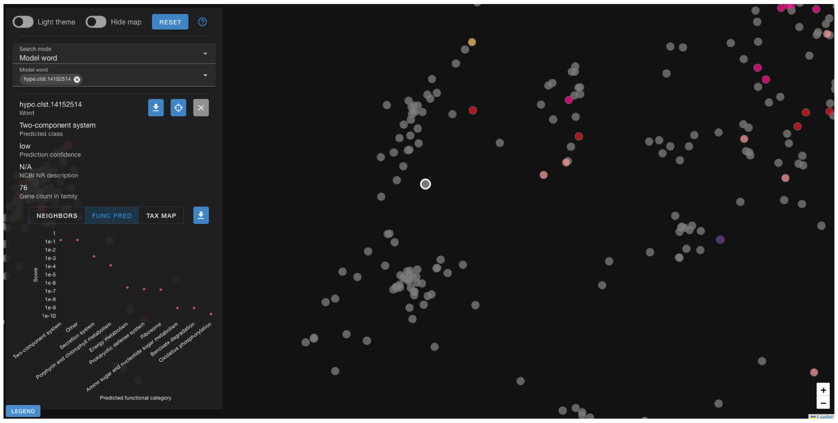

Supplementary Fig. 10: Model word FUNC PRED field (hypo.clst.14152514)

You have the option to download all the results by utilizing the designated download button, including gene family sequences, neighbor information, and predictions. Please take into consideration that the functionality prediction feature (FUNC PRED) is deactivated for known genes, and the taxonomic mapping (TAX MAP) is exclusively accessible for genes that have appeared in the WGS Genomes dataset.

In the **TAX MAP** tab, a graph showcases the taxonomic distribution at the order level for genes obtained from identified organisms within the database. The top 10 orders are displayed, while the remaining categories are consolidated under 'Other.' Additionally, the percentage of genes mapped to a known taxonomy within the entire gene family is provided.

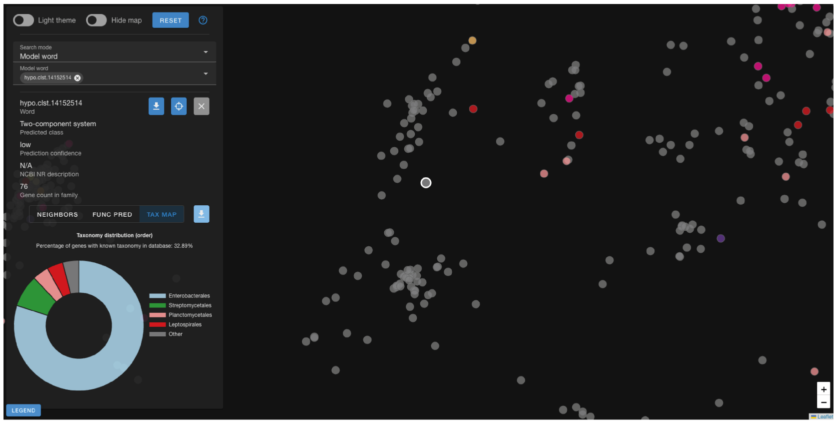

Supplementary Fig. 11: Model word TAX MAP field (hypo.clst.14152514)

Predictive Mode

This mode allows users to submit a sequence query in Fasta format or by direct pasting a protein sequence (with a proceeding >). The web server will provide the predictions for a specific gene or set of genes.

The results will be accessible for download and will be displayed in the information bar on the left. This will encompass essential information about each gene family, along with details regarding the quality of the hit when mapping a sequence to a model word in our database. It is important to note that we only support sequences that exhibit a substantial hit to our database (e-value $< 1e-4$. Sequences that are rare or do not demonstrate a significant hit may not be linked to our resources, as the model was not trained on them. An elaborated case study can be found here: https://github.com/burstein-lab/genomic-nlp-server#getting-started

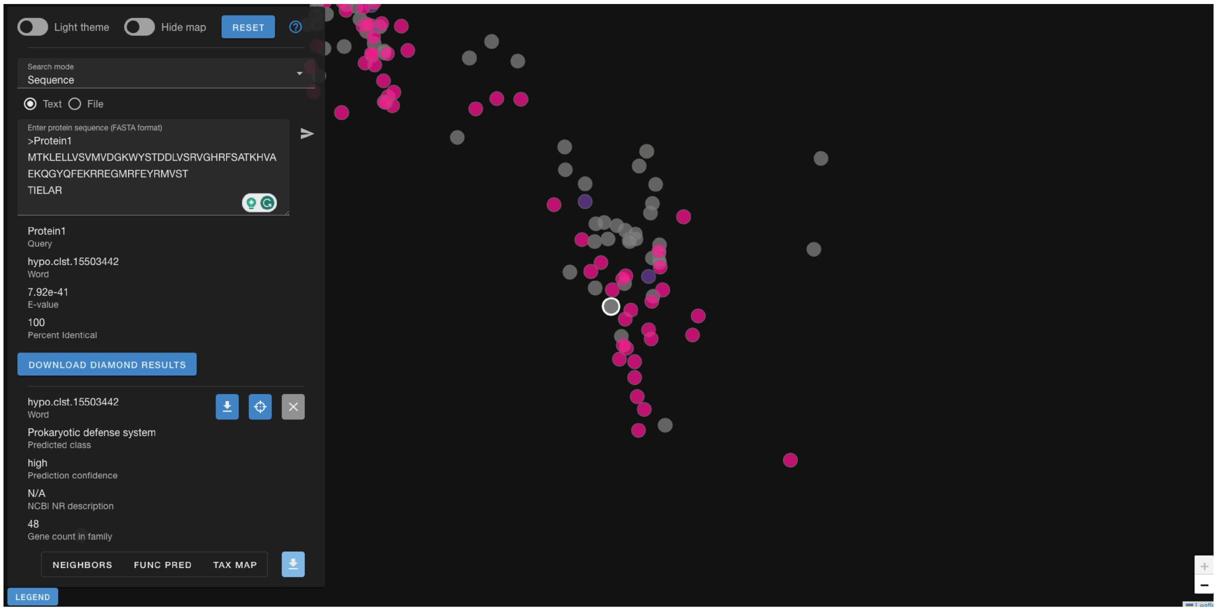

Supplementary Fig. 12: GeNLP prediction mode
